# Enhancing Ishihara and educational images using machine learning: toward accessible learning for colorblind individuals

**DOI:** 10.3389/frai.2025.1676644

**Published:** 2025-10-17

**Authors:** Aahan Ritesh Prajapati, Ajay Goyal

**Affiliations:** ^1^Adani International School, Ahmedabad, India; ^2^Institute of Design, Nirma University, Ahmedabad, India

**Keywords:** Color Vision Deficiency (CVD), protanopia, deuteranopia, Ishihara plates, daltonization, machine learning, LMS cone space, educational image accessibility

## Abstract

Color Vision Deficiency (CVD) affects over 300 million individuals worldwide, with protanopia and deuteranopia being the most common subtypes, causing red–green confusion. This study leverages machine learning to (a) classify reference (considered as normal vision) and simulated protanopia and deuteranopia Ishihara plate images, (b) generate corresponding enhanced versions of these images, and (c) provide improved textbook diagrams (from NCERT books) and other pseudochromatic figures for CVD students, validated through feedback from diagnosed individuals. Tritanopia and milder forms of CVD were excluded in this study. A dataset of 1,400 Ishihara plates was processed to simulate protanopia and deuteranopia perception via standard Red Green Blue (sRGB) to long-, medium-, and short-wavelength cone (LMS) modeling. Enhanced images were generated using a daltonization function defined by the error between reference and simulated images, with enhancement strength (*α*) optimized to maximize contrast gain while minimizing distortion. Feature embeddings from ResNet-50, EfficientNet-B0, and DenseNet-201 were fused and reduced via PCA, followed by One-vs-All (OvA) (classifiers: linear support vector machine, logistic regression, and decision tree), random forest, gradient boosting, and neural network. Results showed optimal enhancement at *α* = 0.54 for deuteranopia and 0.64 for protanopia, achieving contrast gains of 69.6 and 64.3, respectively, with minimal color distortion (ΔE ≈ 4.9) and negligible clipping (<0.002). The OvA strategy achieved 99.7% accuracy, while MLP reached 100% across metrics. Surveys with 15 diagnosed students confirmed substantial perceptual improvement: recognition of previously unreadable digits and symbols increased from <20% to full visibility, with mean ratings above 4/5 for enhanced images. The OvA technique integrated with daltonization can assist in enhancing Ishihara and educational images in real time.

## Introduction

Color Vision Deficiency (CVD) affects approximately 300 million people worldwide (Firth, 2024). It arises from missing cone type, leading to retinal dysfunction and impaired ability to distinguish colors (Brettel et al., 1997; Hunt and Carvalho, 2016). Beyond the biological basis, CVD leads to social and psychological burden, including workplace discrimination, emotional distress, and restricted participation in everyday activities (Barry et al., 2017; Tillem and Gün, 2023).

CVDs are classified by the affected cone class (protan (L), deutan (M), and tritan (S)), each presenting as anomalous trichromacy or, less commonly, dichromacy. Most congenital cases are red–green (protan/deutan), whereas tritan defects are rare (Choi et al., 2019). Due to its simplicity and less classifying time, the Ishihara test is the most widely used classifying method for these CVDs. However, it cannot confirm diagnosis, grade severity, or detect tritanopia.

Motivated by personal experience with red-green color blindness, one of the authors (ARP) and volunteers, under supervision of ophthalmologist (Dr. Nishant Patel, M.B.B.S, Opthalmology & Otorhinolaryngology, D.O.M.S.) and Dr. Shivani Bhatt Charitable Foundation, Anand, Gujarat, conducted an outreach program (between December 2023 to July 2025) with pre-university students across 28 schools in Gujarat, India, classifying more than 10,000 students (Supplementary material S1). This effort identified 121 CVDs those were previously undiagnosed cases, underscoring the scale of unrecognized CVD and the urgent need for accessible real-time classifying methods. Further, the possibilities to provide color friendly NCERT textbook images deeply inspired the authors aimed to reduce academic barriers on CVD students.

Machine learning (ML) models are promising tools for CVD detection and image enhancement or providing color friendly images (Supplementary Tables S2.1, S2.2; Supplementary material 2). Few studies also investigated influence of ambient lighting, type of test, test presentation angle, refractive error correction, and pathological conditions in CVD classifying (Supplementary Tables S2.3; Supplementary material 2). However, limitations remain. Recoloring models often distorts natural color perception, leading to unnatural saturation or elevated ΔE values. Most studies have addressed either classifying or enhancement, with only a few attempts at integrating both (Ananto et al., 2011; Tecson et al., 2017; Pendhari et al., 2024) but with narrow range of image domains. Bile et al. (2023) introduce a non-invasive algorithm that both diagnoses dyschromatopsia and quantifies its severity, and they explicitly position machine learning as the engine for personalized re-education plans. This frames why ML-based enhancement of Ishihara plates is clinically meaningful. Bile (2025) presents the DEA app for color-vision self-re-education, leveraging neuroplasticity with a structured train–test–validate protocol and reporting sustained post-training gains in a severity index (*α*).

This study proposes a ML model in color blindness that first classifies CVD type using Ishihara plate images, then enhances images in realtime based on perceived images generated by removing a cone type from reference images. This classification task mirrors the clinical use of Ishihara plates, where perceiving or failing to perceive the embedded digit constitutes the diagnostic criterion. In this study, reference images represent normal vision and simulated protanopia and deuteranopia images represent deficient perception. By learning image-level features across these groups, the model encodes the same decision boundary used clinically to classify CVD. Thus, ML classification of reference versus simulated images serves to classify the presence and type of CVD. Notably, this is a classifying analogue; confirmation requires further clinical evaluation.

The model was validated by enhancing textbook diagrams and multiple variants of Ishihara plate (single-digit, double-digit, animal, and symbolic forms). To strengthen the results, two detailed feedback were done with CVD individuals. To identify best suitable ML model, this study applied and compared various ML techniques. This study targets classroom material, aiming to reduce the educational barriers faced by young students with CVD.

In this study we restrict modeling and evaluation to red–green dichromacy (protanopia, deuteranopia); anomalous trichromacy (protanomaly, deuteranomaly) is out of scope and slated for future work. Also, given the constraints of Ishihara-based classifying, Tritanopia, partial anomalies, and severity grading were excluded. By laying a methodological foundation, this work contributes toward future assistive devices capable of real-time classifying and enhancement of pseudochromatic images and textbook images for students.

## Methodology

**Figure 1:**
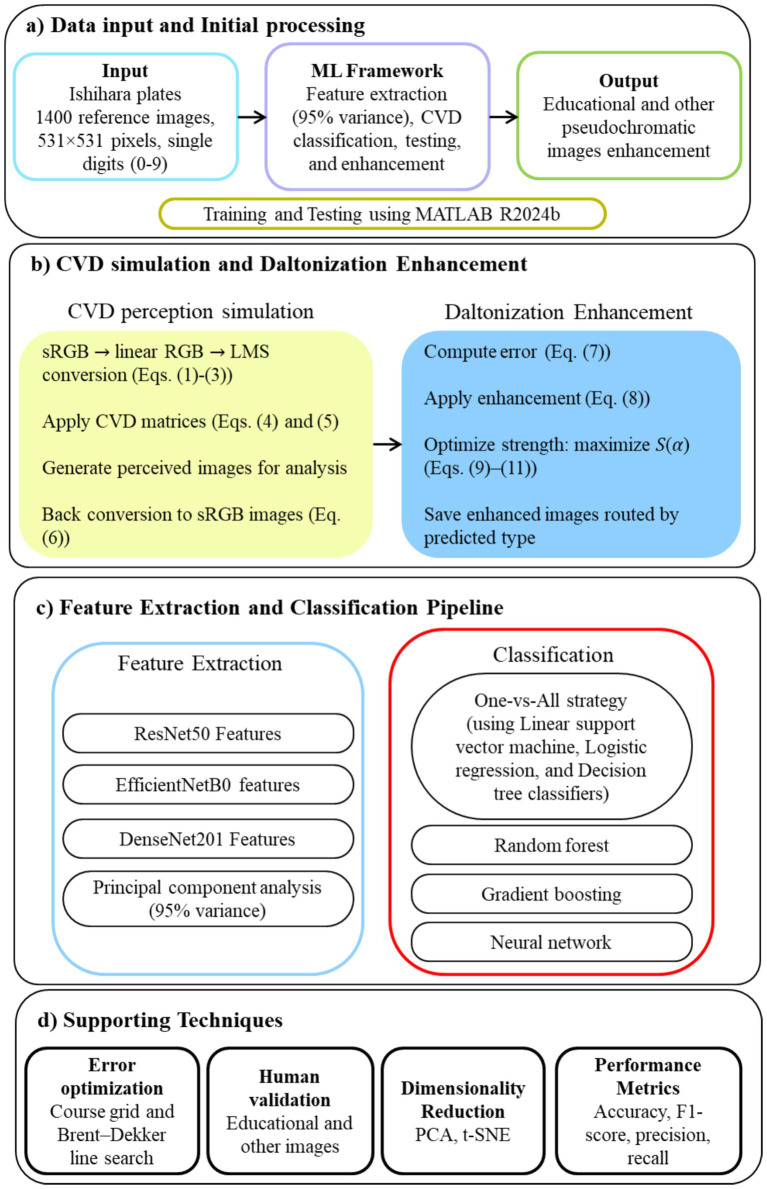
Architecture and workflow of the proposed CVD–ML system.

Figure 1 shows the model architecture of the proposed study. All steps were coded in MATLAB R2024b (The MathWorks Inc., Natick, Massachusetts). This study received ethical clearance from the Institutional Ethics Committee of Nirma University, Ahmedabad, India (Certificate No. IEC/NU/25/ID/01) dated May 26, 2025. Written consent was obtained from fifteen available CVD individuals (eight deuteranopia individuals) who participated in the survey. All participants were diagnosed with either deuteranopia or protanopia.

### Dataset

A dataset of 1,400 reference Ishihara images (531 × 531 pixels) ( $I_{\text{sRGB}}$ ) was adopted from the Kaggle repository.^1^ Each image contains a single-digit numeral (0–9), embedded within a stochastically distributed pattern of polychromatic dots with selective chromatic contrast designed to challenge color perception. This open-source dataset was originally generated at Kuban State University. These images are not patient-derived diagnostic records but computer-generated Ishihara-style plates, generated using combinations of Google fonts, digits, and color schemes.

### Generating perceived and enhanced images

Each reference image $I_{\text{sRGB}}$ in the standard Red-Green-Blue (sRGB) space was first converted to linear RGB ( $I_{\mathrm{lin}}$ ) using the IEC sRGB opto-electronic transfer function Equation 1 (IEC 61966-2-1, 1999). s is the normalized sRGB channel value for a given pixel.


(1) $$I_{\mathrm{lin}}(s)=\{\begin{array}{c} \frac{s}{12.92}\;\;s\leq 0.04045\\ {\left(\frac{s+0.055}{1.055}\right)}^{2.4},\;s>0.0405 \end{array}$$

Linear RGB values were transformed to LMS cone response (Equations 2, 3) (Machado et al., 2009). sRGB was converted to LMS because device RGB encodes monitor primaries, not the human visual sensors; cone space provides a physiologically grounded basis for both simulating dyschromatopsia and designing compensations (Hunt and Pointer, 2011). Deficiency simulation and compensation follow established LMS models. All deficiency and enhancement operations are performed in cone space and in opponent coordinates so that edits target the channels actually affected in CVD type conditions rather than entangling device-dependent RGB axes (Stockman and Sharpe, 2000).


(2) $$\mathrm{LMS}=M_{\mathrm{RGB}\to \mathrm{LMS}}.\mathrm{RG}B_{\mathrm{lin}}$$

(3) $$M_{\mathrm{RGB}\to \mathrm{LMS}}=[\begin{array}{ccc} 0.314 & 0.639 & 0.046\\ 0.155 & 0.757 & 0.087\\ 0.018 & 0.109 & 0.872 \end{array}]$$

CVD perception was simulated using deficiency-specific matrices $M_{\mathrm{De}}$ / $M_{\mathrm{Pr}}\;$ (Machado et al., 2009) (Equations 4, 5) (Machado et al., 2009).


(4) $$M_{\mathrm{De}}=[\begin{array}{ccc} 0.625 & 0.375 & 0.000\\ 0.700 & 0.300 & 0.000\\ 0.000 & 0.300 & 0.700 \end{array}]$$

(5) $$M_{\mathrm{Pr}}=[\begin{array}{ccc} 0.567 & 0.433 & 0.000\\ 0.558 & 0.442 & 0.000\\ 0.000 & 0.242 & 0.758 \end{array}]$$

These matrices projected the LMS responses into the deficient observer’s perceptual space. The simulated LMS values were converted back to linear RGB ( $M_{\mathrm{LMS}\to \mathrm{RGB}}={M_{\mathrm{RGB}\to \mathrm{LMS}}}^{-1}$ ), and then reconverted to sRGB using the inverse gamma function (Equation 6) (IEC 61966-2-1, 1999). l is the linear-light channel value.


(6) $$\text{sRGB}(l)=\{\begin{array}{c} 12.92\;l\;\;l\leq 0.0031308\\ 1.055l^{\frac{1}{2.4}}-0.055,\;l>0.0031308 \end{array}$$

This yielded how the reference plate would appear to an observer with the corresponding CVD. These simulated images were saved in separate folders: Perceived_Deuteranopia and Perceived_Protanopia. Labels 0, 1, and 2 were assigned to reference images (assumed to be images for normal vision), perceived deuteranopia, and perceived protanopia images across the dataset. All images generated from a given plate, whether reference or simulated, were assigned exclusively to either the training set (80% of the dataset) or the testing set.

An optimized daltonization framework was applied to correct lost chromatic cues while maintaining perceptual naturalness to generate enhanced images. The error signal ( E ) was computed in sRGB space (Equations 7) (Farup, 2020). Equation 7 is the standard way to measure “what information was lost” when a color-normal observer’s plate is viewed through a simulated CVD transform (Farup, 2020). The enhanced images ( $I_{\mathrm{enh}}$ ) were obtained using Equation 8 (Simon-Liedtke and Farup, 2018). α∈[0,1.5] (Simon-Liedtke and Farup, 2018) is an enhancement strength parameter. clip(.,0,1) ensures all channel values remain in the displayable range(Simon-Liedtke and Farup, 2018). Equation (8) reinjects that lost signal with α . This linear add-back is the design goal of daltonization: amplify contrasts along confusion directions while leaving everything else as untouched as possible. The explicit clip operator is a perceptual safeguard that prevents out-of-gamut excursions from creating hue or lightness artifacts on real displays (Simon-Liedtke and Farup, 2018).


(7) $$E=I_{\text{sRGB}}-I_{\mathrm{def}}$$

(8) $$I_{\mathrm{enh}}=\text{clip}(I_{\text{sRGB}}+\alpha .E,0,1)$$

The optimal $\alpha^{*}$ for each CVD type (protanopia and deuteranopia) was determined by maximizing an objective function (Equation 9) (Simon-Liedtke and Farup, 2018). $\Delta C_{\mathrm{CVD}}$ is the change in contrast under simulated CVD, computed using Equation 10 (Simon-Liedtke and Farup, 2018). $L_{k}$ denotes the CIELAB $L^{*}a^{*}b^{*}$ channels of the simulated images. $\Delta E_{\mathrm{norm}}$ is the mean CIE76 color difference between original and enhanced images under normal vision, computed using Equation 11 (Simon-Liedtke and Farup, 2018). N is the number of pixels in the image. $f_{\text{clip}}$ is the fraction of pixels with at least one channel at 0 or 1 after enhancement. $\lambda_{\mathrm{nat}}=0.15$ and $\beta_{\text{clip}}=0.05$ are penalty weights controlling the tradeoff between perceptual naturalness and avoidance of channel saturation.


(9) $$S(\alpha )=\Delta C_{\mathrm{CVD}}-\lambda_{\mathrm{nat}}\Delta E_{\mathrm{norm}}-\beta_{\text{clip}}f_{\text{clip}}$$

(10) $$\Delta C_{\mathrm{CVD}}=[\sum_{k=1}^{3}\mathrm{Var}(L_{k}^{\text{corr}})]-[\sum_{k=1}^{3}\mathrm{Var}(L_{k}^{\text{orig}})]$$

(11) $$\Delta E_{\mathrm{norm}}=\frac{1}{N}\sum_{p=1}^{N}\sqrt{{\left(\Delta L_{p}^{*}\right)}^{2}+{\left(\Delta a_{p}^{*}\right)}^{2}+{\left(\Delta b_{p}^{*}\right)}^{2}}$$

S(α) was evaluated over a fixed grid α∈{0.3,0.35,….1.5} . Then, Brent-Dekker bounded minimization was applied to evaluate - S(α) within the interval of 0 to 1.5. The resulting $\alpha_{\mathrm{Pr}}^{*}$ and $\alpha_{\mathrm{De}}^{*}$ were applied to the full dataset to produce two enhanced images, each specifically optimized for one CVD type.

The objective S(α) in Equation 9 formalizes the trade-off the viewer actually experiences. The first term, ΔC from Equation 10, measures how much useful structure becomes more legible to a CVD observer after enhancement by comparing channel variances in the simulated CIELAB image before and after correction; higher is better because it means more separable cues where the deficiency collapses chromatic differences (Simon-Liedtke and Farup, 2018). The second term, $\Delta E_{\mathrm{norm}}$ (Equation 11), pushes back against heavy-handed edits by penalizing average CIE76 color differences under normal vision; lower is better because improvements are required for CVD observers without making the image look “wrong” to everyone else (Simon-Liedtke and Farup, 2018). The third term, $f_{\text{clip}}$ , directly penalizes saturation hits so the optimizer prefers solutions that stay inside the display gamut most of the time, which in practice reduces banding and haloing on high-contrast dot boundaries (Simon-Liedtke and Farup, 2018). The weights $\lambda_{\mathrm{nat}}$ and $\beta_{\text{clip}}$ express design priorities: readable contrasts for CVD viewers first, with focus to keep naturalness and avoid clipping. Coarse grid over α ∈[0.3,1.5] followed by Brent-Dekker minimization on −S(α) is a practical way to find $\alpha_{\mathrm{Pr}}^{*}$ and $\alpha_{\mathrm{De}}^{*}$ robustly. It is fast, reproducible, and avoids the brittleness of gradient-based methods on non-smooth objectives with a clipping term (Simon-Liedtke and Farup, 2018; Farup, 2020). Derivation of Equations 1–11 are given in Supplementary material 3.

### Feature extraction

Feature extraction was performed using three convolutional neural network (CNN) backbones pretrained on ImageNet: ResNet-50 (2048-dimensional features ( $X_{\mathrm{RN}50}$ ) from the average pooling layer) (He et al., 2015), EfficientNet-B0 (1280-dimensional global average pooling features ( $X_{\mathrm{ENB}0}$ )) (Tan and Le, 2020), and DenseNet-201 (1920-dimensional features ( $X_{\mathrm{DN}201}$ ) from the average pooling layer) (Huang et al., 2018). ResNet’s residual blocks emphasize mid−/high-level edge and shape cues; EfficientNet’s compound scaling maintains fine local structure at modest capacity; DenseNet’s dense connectivity promotes feature reuse and stable gradients, preserving subtle dot-pattern statistics. Each network processed resized images matching its native input resolution, and deep embeddings were obtained via feed-forward inference. The embeddings from the three architectures were concatenated to form a fused feature vector ( $X_{\text{fused}}$ ) (Equation 12) (Li et al., 2025), resulting in a 5,248-dimensional feature space for each image.


(12) $$X_{\text{fused}}=[X_{\mathrm{RN}50}\;\|\;X_{\mathrm{ENB}0}\;\|\;X_{\mathrm{DN}201}]$$

The training subset underwent column-wise z-score normalization (Equation13) (Tryon et al., 2025). $\mu_{j}$ and $\sigma_{j}$ are the mean and standard deviation of feature j in the training set.


(13) $$X_{\mathrm{ij}}^{\prime }=\frac{X_{\mathrm{ij}}-\mu_{j}}{\sigma_{j}}$$

Dimensionality reduction was conducted using principal component analysis (PCA) (Jolliffe and Cadima, 2016) on the normalized training set to retain a standard target variance of 95%. The transformation was then applied to the validation set using Equation 14 (Mochurad et al., 2025). $W_{\mathrm{PCA}}$ contains the top principal components computed to meet a 95% variance threshold.


(14) $$X_{\mathrm{PCA}}=(X\prime -\mu_{\mathrm{PCA}}).W_{\mathrm{PCA}}$$

This design was guided by five criteria: (1) architectural diversity, established by combining embeddings from three structurally distinct CNN backbones; (2) descriptors derived from ImageNet-pretrained networks, which are not explicitly optimized for chromatic opponency, but were sanity-checked on recolored probes to confirm retention of coarse color variations, with fusion across architectures used to mitigate the risk of losing finer chromatic cues; (3) dimensionality control via PCA at a 95% variance threshold, chosen as a pragmatic balance between tractability and information preservation, with sensitivity checks at alternative cutoffs confirming stable performance; (4) a non-inferiority safeguard requiring the fused vector’s cross-validated macro-AUC to remain within a practically defined margin of the strongest backbone, recognizing that margins smaller than inter-fold variability are interpreted heuristically rather than as strict statistical bounds; and (5) reproducibility through fixed preprocessing statistics (*μ*, *σ*, PCA transform), frozen pretrained weights, consistent resizing and normalization to ensure repeatability under controlled environments.

### Classification model for diagnosis

A One-vs-All (OvA) classification model (Hashemi et al., 2009; Lutu and Engelbrecht, 2012; Yan et al., 2020; Kumar et al., 2024) was trained to classify perceived De, and Pr, and the reference Ishihara plate. Three distinct classifiers were trained for each OvA task: linear support vector machine (SVM) (Varghese and Gopan, 2020), logistic regression (Manasa et al., 2024), and decision tree (Ramkumar and Sivaprakash, 2025). In addition, random forest (Varghese and Gopan, 2020), gradient boosting, radial basis function SVM (SVM-RBF) (Varghese and Gopan, 2020), and a shallow multilayer perceptron (MLP) (Tian et al., 2004) were used to compare the results. Class-wise accuracy, precision, recall, and F1 score were computed (Sisodia et al., 2023). The enhancement algorithm was integrated with the One-vs-All model to display an enhanced image based on the classification classifying result for the test set or validation images.

### Validation dataset: enhancing educational and other pseudochromatic images

**Figure 2:**
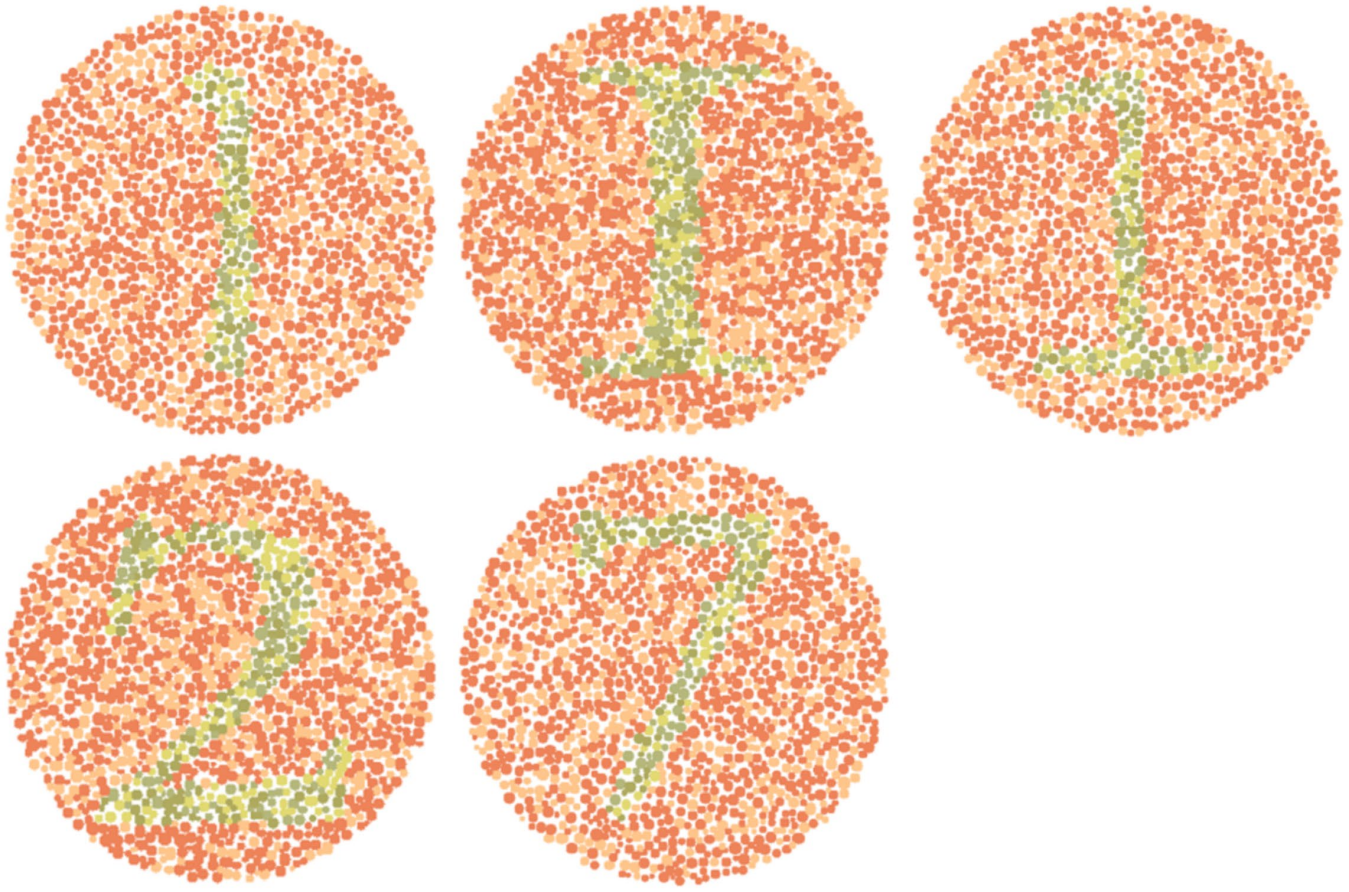
Reference images from dataset with high chances of red-green color confusion.

Based on the results, two subsequent in-person surveys were conducted with fifteen and then eleven 9th- to 11th-grade students of D.N. High School suffering from either De or Pr. In particular, five reference images from the dataset (with a subsequent enhanced image placed to its right) with the highest possibilities of red-green confusion were shown to these students (Figure 2). These images (numbered 153, 157, 253, 297, and 1,101) were identified based on the maximum Δ E between reference and corresponding simulated images. Students were asked to compare these two groups of five images, and then they responded to various open-ended, single, multiple-choice, or rating-based questions.

**Figure 3:**
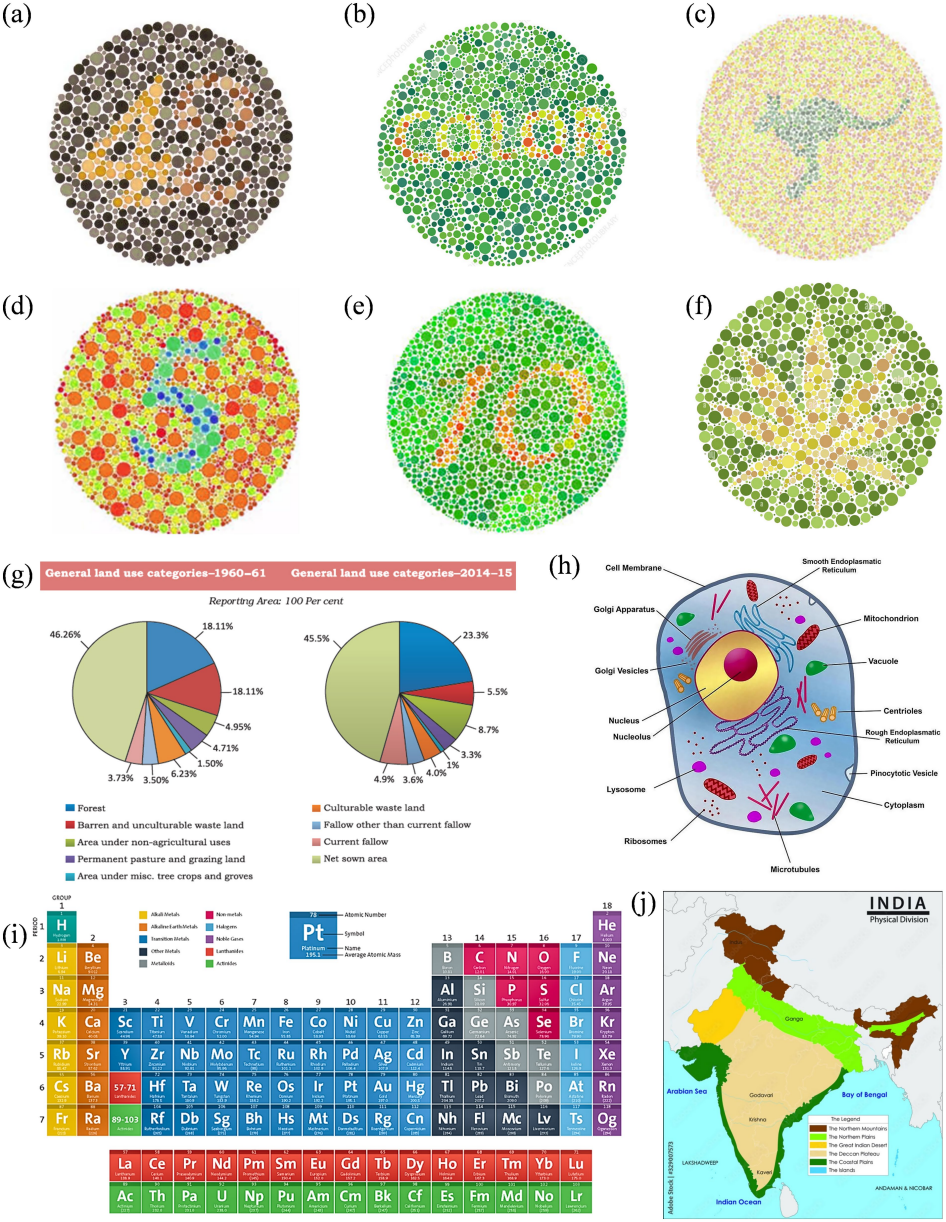
Reference set of images representing normal color vision, selected for subsequent enhancement targeting Deuteranopia and Protanopia: **(a–f)** Ishihara plates designed with varying content: numerals **(a,d,e)**, alphabetic word **(b)**, animal profile (kangaroo) **(c)**, and abstract leaf shape **(f)**; **(g)** pie charts comparing land use in India between 1960–61 and 2014–15; **(h)** a labeled eukaryotic cell diagram; **(i)** periodic table grouped by element categories; and **(j)** physical map of India showing key terrain types.

Based on the results, another survey included more specific questions. In this survey, other images were also included (enhanced using the same algorithm). These include two-digit Ishihara images, similar images with a leaf and an animal surrounded by colored dots, a periodic table, a physical map of India, and a eukaryotic cell. These images were adopted from open-source or purchased from professional websites (Planemad, 2024; American Chemical Society, 2025; Radu, 2025). Reference normal vision images are given in Figure 3. Participants were asked to check the clarity, interpretability, and usability of enhanced images. Responses were collected using closed-format questions (e.g., Likert ratings, forced-choice selection) and short written feedback. Participants also identified image types where enhancements were most or least helpful and indicated whether such improvements should be applied in textbooks. The survey was given in English and was designed to be completed in 15–20 min.

## Results

### ML performance evaluation

Deuteranopia reached its optimum at *α* = 0.54, scoring 68.84 and contrast improvement (ΔC) of 69.57. For protanopia, α, score and ΔC was 0.64, 63.53 and 64.28, respectively. In both cases, color fidelity was maintained (ΔE ≈ 4.9) with negligible clipping (<0.002). Table 1 shows the confusion matrix of OvA technique for classifier labels (0: reference image, 1: Deuteranopia and 2: Protanopia) in the trained and tested dataset. The OvA confusion matrices show near-perfect separation: in training, 3,349 of 3,360 images are correctly classified, with only class-1 leaking slightly into classes 0 (1 case) and 2 (7 cases). On the test set, 839 of 840 images are correct; the one error is a class-1 image predicted as class-2. Table 2 shows the performance matrices of trained and tested dataset of the OvA model for each CVD classifier. Per-class metrics confirm uniformly high performance: training precision/recall/F1 are ≥0.994 across all classes, with minor leakage only for class-1 (8 FN, 3 FP) and class-2 (3 FN, 7 FP). On the test set, all classes are essentially perfect (F1 = 0.998–1.000); the only slips are a single FN for class-1 and one FP against class-2, keeping class-wise accuracy at 0.999–1.000. Table 3 shows the comparison of different machine learning models based on classification performance metrics. All models perform strongly, with MLP achieving perfect scores (1.000) and SVM-RBF/OvA close behind at 0.997 across all metrics. GradBoost (0.993) and Random Forest (0.992) trail slightly but remain high.

**Table 1:** Confusion matrix showing correct and misclassified instances by OvA technique for classifier labels in the trained and tested dataset.

Labels	0	1	2
Trained			
0	1,132	0	0
1	1	1,102	7
2	0	3	1,115
Tested			
0	282	0	0
1	0	282	1
2	0	0	275

**Table 2:** Performance matrices of trained and tested dataset of the OvA model for each CVD classifier.

Class	TP	FP	FN	Accuracy	Precision	Recall	F1-score
Trained dataset							
0	1,132	1	0	1.000	0.999	1.000	1.000
1	1,102	3	8	0.997	0.997	0.993	0.995
2	1,115	7	3	0.997	0.994	0.997	0.996
Tested dataset							
0	282	0	0	1.000	1.000	1.000	1.000
1	282	0	1	0.999	1.000	0.997	0.998
2	275	1	0	0.999	0.996	1.000	0.998

**Table 3:** Comparison of different machine learning models based on classification performance metrics.

Model	Accuracy	Precision	Recall	F1 score
OvA strategy	0.997	0.997	0.997	0.997
Random Forest	0.992	0.992	0.992	0.992
GradBoost	0.993	0.993	0.993	0.993
SVM-RBF	0.997	0.997	0.997	0.997
MLP	1.000	1.000	1.000	1.000

**Figure 4:**
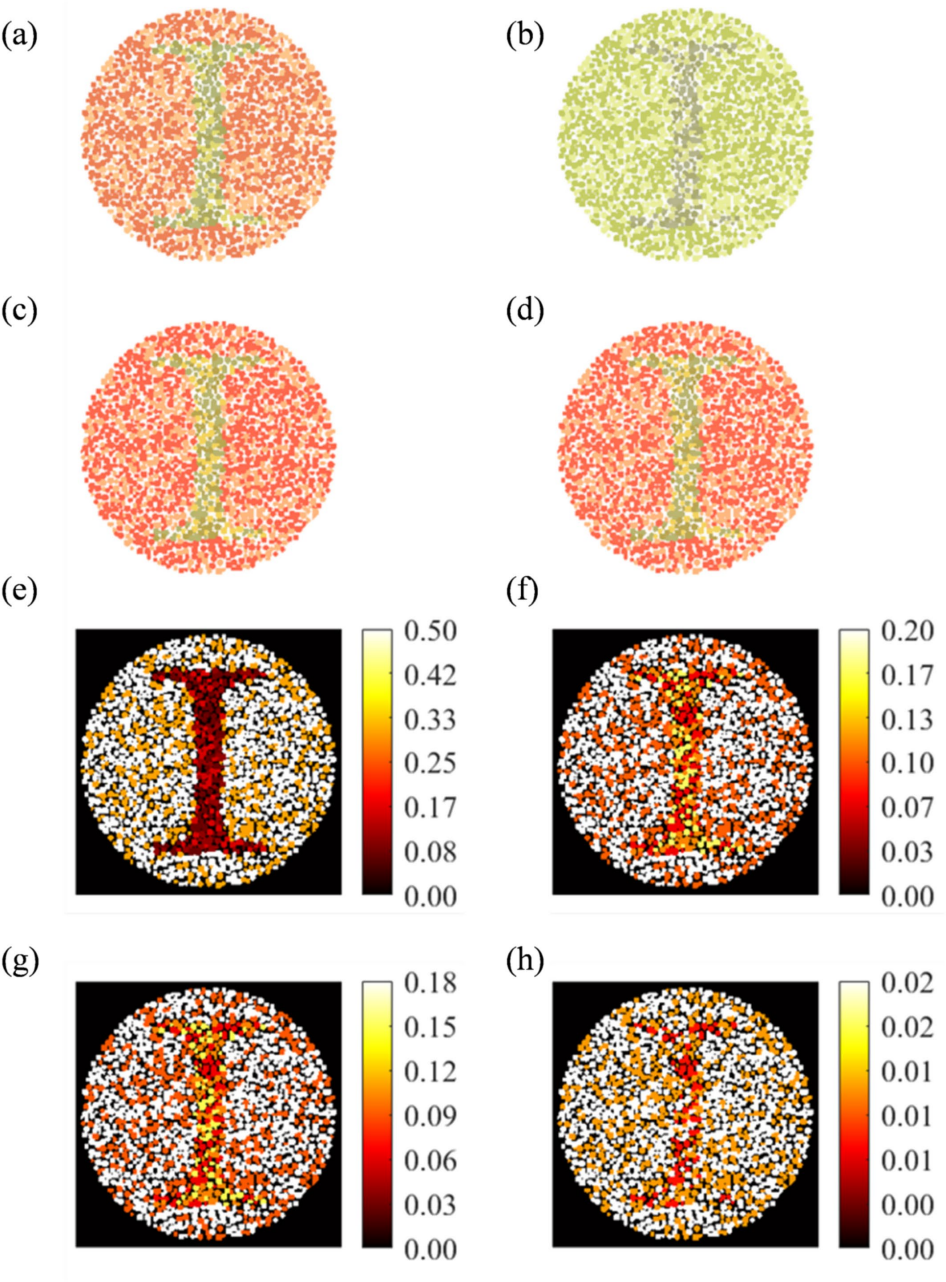
Analysis of wrongly diagnosed image number 157 for Deuteranopia CVD patient: **(a)** Original, **(b)** actual perceived, **(c)** actual enhanced, and **(d)** Protanopia diagnosed enhanced image. RGB difference heat map: **(e: a,b), (f: a,c), (g: a,d)**, and **(h: c, d)**.

Figure 4 shows the results of a misclassified (image number 157) deuteranopia. Figures 4a–c show the original, perceived, and enhanced image with “1” digit, respectively. The model diagnosed image as protanopia, whose enhanced image is shown in Figure 4d. The RGB heat maps between the original vs. perceived difference (Figure 4e) showed a maximum distortion difference of 0.50. Heat map of reference versus deuteranopia-enhanced (Figure 4f) and reference versus protanopia-enhanced (Figure 4g) showed a maximum enhancement of 0.20 and 0.18, respectively. The two enhanced images showed no significant color difference (Figure 4h).

### Survey-based visualization of enhanced images

**Figure 5:**
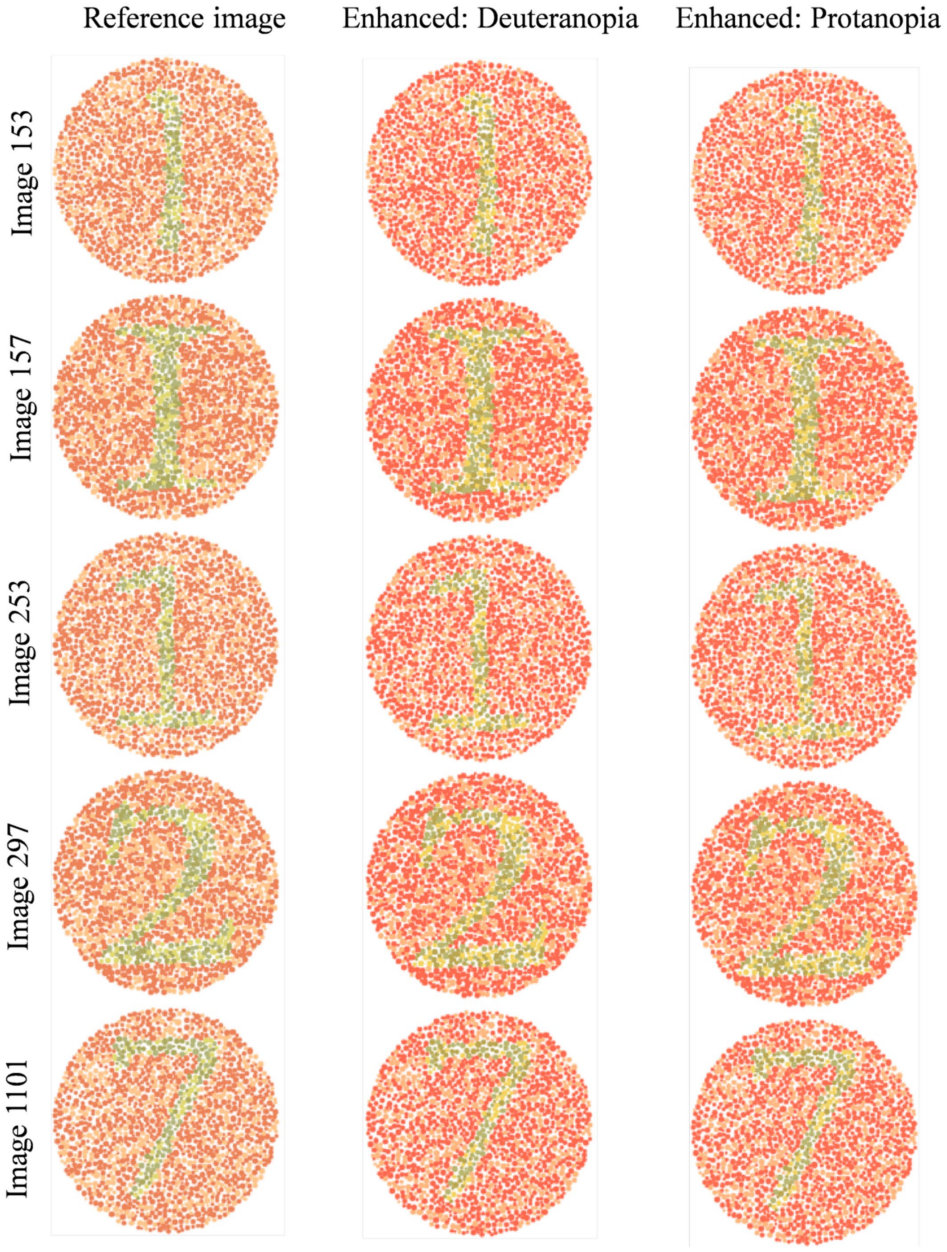
Visual comparison of enhanced images with references Ishihara plates shown in Figure 2.

Figure 5 shows normal vision (Figure 2) and corresponding enhanced Ishihara plates with high chances of red-green confusion in simulated 1,400 images.

Image 153 showed complete agreement: all eleven participants preferred the enhanced image. Recognition of the digit “1” improved from minimal detectability in the reference (De: 2/6; Pr: 1/5) to complete recognition in the enhanced image (De: 6/6; Pr: 5/5). Structural details such as the horizontal line were visible to only 1–2 participants in the reference, but to 6 in the enhanced version. The mean improvement ratings (out of 5) were 4.08 ± 0.64 (De) and 4.2 ± 0.45 (Pr).

Image 157 followed a similar pattern. None of the eleven participants could identify the alphabet “I” in the reference; five De and four Pr participants recognized it in the enhanced image. The horizontal line improved from near-invisibility (1/6 De, 1/5 Pr) to full detectability in both groups. Textbook inclusion preference was strong, with 10/11 participants affirming they would rely on the enhanced version. Mean improvement scores were 4.08 (De) and 4.0 ± 0.63 (Pr).

Image 253 presented slightly more variation. Digit recognition in the reference remained low (De: 1/6; Pr: 1/5), but was restored in the enhanced image (De: 6/6; Pr: 5/5). Line visibility improved similarly (De: 1–5; Pr: 1–5). The average scores was 4.4 ± 0.55 (Pr) and a broader 2–5 range in De responses.

Image 297 responses introduced slight perceptual ambiguity. All protanopes and five deuteranopes preferred the enhanced image. One deuteranope considered both images equally helpful. Digit “2” was difficult to identify in the reference (De: 2/6; Pr: 1/5), but was recognized instantly by all eleven in the enhanced version. Visibility of the upper curve improved from partial detectability (mostly “Maybe” responses) to complete recognition across both groups. Improvement scores were 4.1 ± 0.48 (Pr) and more variable in De (range: 2–5), possibly influenced by prior familiarity or reduced baseline distortion.

For image 1,101, five De and all five Pr selected the enhanced version as most helpful. Digit “7” recognition improved from 2/6 (De) and 1/5 (Pr) in the reference to 5/6 and 5/5, respectively, in the enhanced image. The junction between horizontal and vertical lines was a key differentiator, which was visible to all in the enhanced version but detected by only 1–2 participants per group in the reference image.

**Figure 6:**
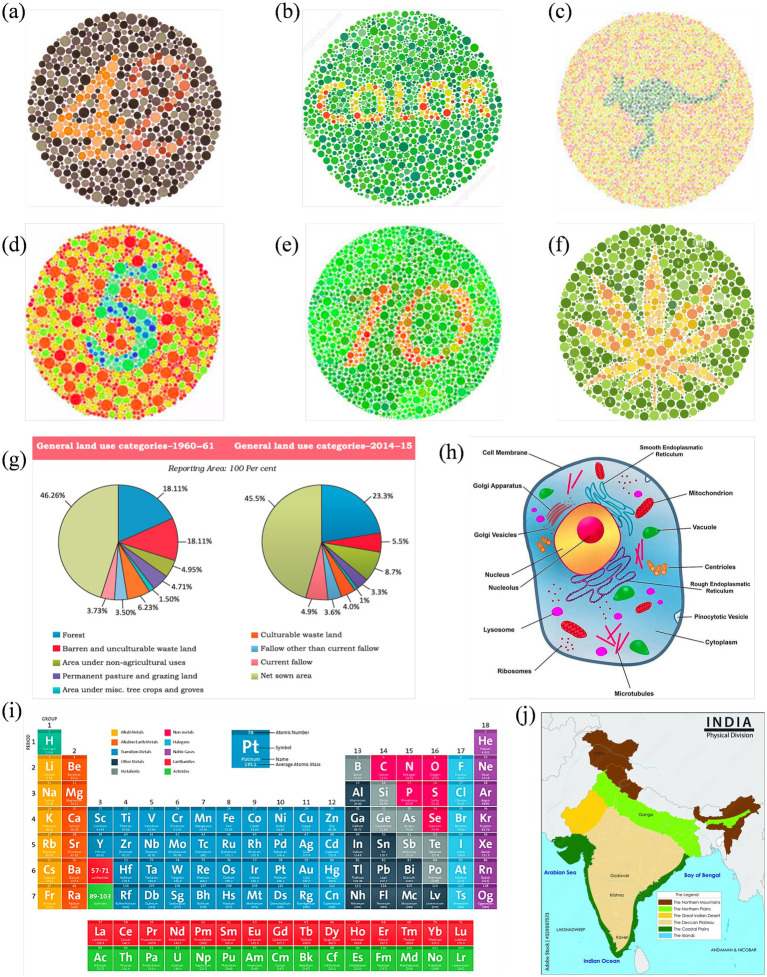
Enhanced image set optimized for deuteranopia, corresponding to the reference visuals in Figure 3.

**Figure 7:**
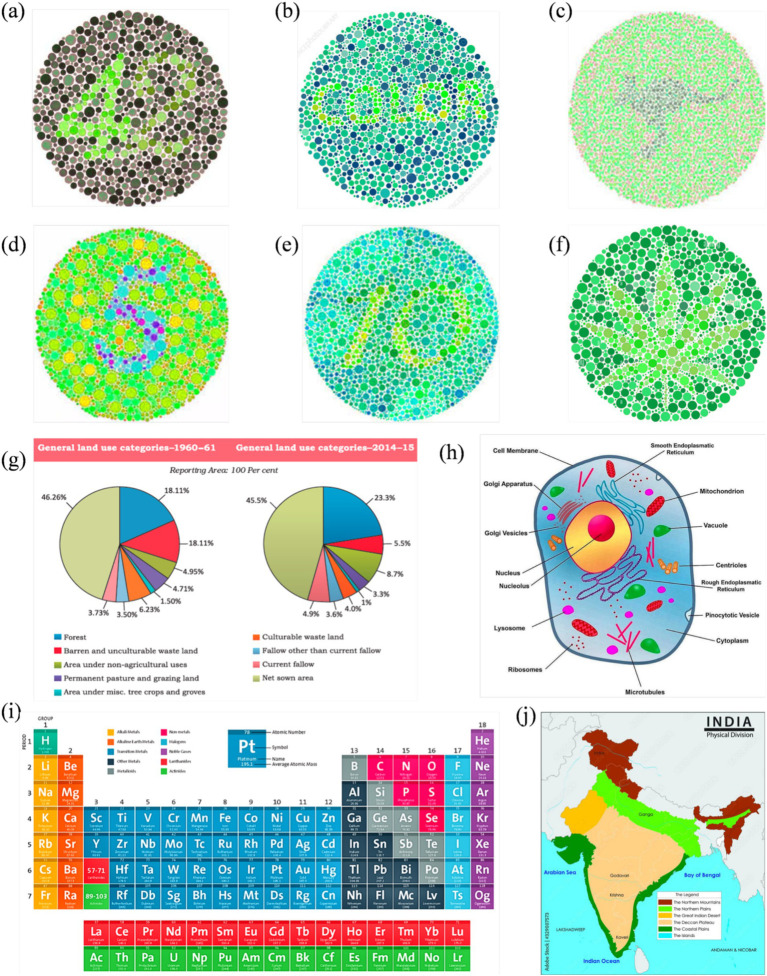
Enhanced image set optimized for protanopia, corresponding to the reference visuals in Figure 3.

Figures 6, 7 show enhanced images for De and Pr, respectively, of the reference images shown in Figure 3. Figure 3a (digits “4” and “2”) was the strongest case of convergence: both groups reported substantial improvement with the enhanced version. All protanopes and four of six deuteranopes selected the enhanced image as most helpful. Digit recognition rose from 2/6 (De) and 1/5 (Pr) to complete recognition in the enhanced version across both groups. Structural features, such as the junction of “4” and curvature of “2,” were visible in the enhanced version for all participants. Additional visibility of digit “7” in the enhanced image was confirmed across all protanopes, but yielded mixed results in deuteranopes. Average improvement scores were 3.25 (De) and 4.2 (Pr).

Figure 3b (text “COLOR”) produced a consistent benefit for deuteranopes, but a divided response among protanopes. While five deuteranopes preferred the enhanced image, protanopes were split (3 vs. 2). Word recognition in the reference image was poor for deuteranopes (1/6) and strong for protanopes (4/5). The enhanced version improved recognition to 6/6 (De) but slightly declined among protanopes (3/5). Both groups showed mixed though positive results for clarity.

Figure 3c (text and silhouette of “kangaroo”) was the least effective image overall. Both groups showed a neutral response to enhancement. 3/6 deuteranopes and 3/5 protanopes preferred the enhanced version, but the visibility of the word “kangaroo” and animal shape was inconsistent. Among protanopes, only 2/5 saw the animal in the enhanced image, compared to 4/5 in the reference image. Deuteranopes reported better silhouette clarity in the enhanced image but noted the reference image was already legible. Mean scores were 3.3 (De) and 2.6 (Pr), indicating a need for content-specific tuning in organic figure enhancements.

Figure 3d (digit “5”) was a strong performer across both groups. All protanopes and deuteranopes favored the enhanced image. Digit recognition increased from 3/6 (De) and 0/5 (Pr) in the reference image to 6/6 and 5/5 in the enhanced versions, respectively. Visibility of curved segments improved to full clarity in both groups, confirming that edge enhancement and contrast sharpening effectively resolved numeric ambiguity. Average ratings were 4.0 for both CVDs.

Figure 3e (digits “1” and “0”) showed that all deuteranopes and three protanopes preferred the enhanced image. Recognition of digits improved from 1/6 (De) and 2/5 (Pr) in the reference to 5/6 and 2/5 in the enhanced image, respectively. For both digit “1” line junctions and “0” curvature, enhanced images offered clearer visibility to most, but not all. Ratings were 3.8 (Pr) and 4 (De).

Figure 3f (flower petals) demonstrated reliable gains in both groups, especially for shape detection. All protanopes and five deuteranopes selected the enhanced image as most helpful. Petal recognition improved from 2/6 (De) and 2/5 (Pr) in the reference to full clarity in the enhanced version.

Figure 3g (Land Use Pie Chart) showed complete category-level distinction between reference and enhanced images across both CVD groups. All participants successfully differentiated red vs. green, pink vs. orange, and blue vs. purple zones in reference and enhanced images. However, five deuteranopes and all five protanopes preferred enhanced image, citing improved comfort, darker tones, and reduced cognitive effort. The enhancement was favored for classroom applicability across both groups.

Figure 3h (Physical Map of India) enabled perfect region-level color distinction in both groups. All participants in both groups distinguished landform zones and identified river names with no reported issues. Text clarity improved in the enhanced image for deuteranopes (5/6), while protanopes reported equal legibility across both versions.

Figure 3i (Periodic Table) was interpreted successfully by all participants in both groups, with clear differentiation of Non-metals and Lanthanides (where color confusion was expected). However, all participants rated the enhanced image higher in clarity, with protanopes assigning consistent ratings of 4/5 and deuteranopes averaging 4.0.

Figure 3j (Labeled Eukaryotic Cell) demonstrated a stronger enhancement effect for protanopes. Four of five protanopes and four of six deuteranopes preferred the enhanced image for differentiating Vacuole and Microtubules (where color confusion was expected). Deuteranopes additionally rated the enhanced image high in color contrast and shape clarity (5/6 selected “color contrast” as the key improvement).

## Discussion

The findings of this study demonstrate that machine learning coupled with optimized daltonization can classify for red-green CVD and considerably improve the accessibility of educational images. The OvA strategy performance confirmed its ability to differentiate deuteranopia, protanopia, and normal vision images robustly. AlEssa and Alzahrani (2024) achieved >90% using an EEG-SVM system, Pendhari et al. (2024) reported upto 98% with CNNs, and Akalın et al. (2025) exceeded 90% with a MobileNet evaluator.

Comparison with other classifiers further clarifies the tradeoffs. MLP achieved perfect scores across all metrics, suggests risk of overfitting. By contrast, OvA delivered almost identical performance with better transparency, distributing decision boundaries across simpler binary tasks. SVM with RBF kernel also achieved 99.7% accuracy. Tree-based models such as Random Forest and Gradient Boosting performed reliably (99.2–99.3%).

Optimal *α* values produced strong contrast gains of ΔC = 69.6 (deuteranopia) and 64.3 (protanopia), with minimal perceptual distortion (ΔE ≈ 4.9) and negligible clipping. This demonstrates that color cues lost in CVD perception can be selectively recovered while maintaining naturalness. Importantly, these quantitative improvements translated into real perceptual benefits. In controlled surveys with CVD affected students, recognition of digits, alphabets, and structural features improved. Images that were previously unreadable, for example, Image 153, where digits were nearly invisible, became fully legible across all participants after enhancement. On multiple plates, digit recognition improved from fewer than 2 of 11 participants to complete recognition. Structural clarity, such as line junctions or curves, was consistently restored. Preference rating for enhanced images was high in most validated figures across deuteranopes and protanopes, with mean scores often above four on a 5-point scale.

The validation extended beyond Ishihara-like plates. Enhanced classroom images, including periodic tables, maps, pie charts, and biological diagrams, were rated as more interpretable and usable. Physical map of India and the pie chart achieved unanimous preference for the enhanced versions, with participants reporting reduced effort and clearer region boundaries. The periodic table and cell diagram showed targeted benefits in areas of known red-green confusion. These outcomes confirm that the enhancement algorithm is technically effective and practically valuable in educational settings, where misinterpretation of diagrams can hinder learning.

The survey also highlighted variability, reflecting the nuanced nature of visual perception in CVD. Certain images, such as the kangaroo silhouette, showed neutral or mixed responses, and in some cases, enhancements benefited deuteranopes more than protanopes. This suggests that content-specific optimization may be necessary, or a consequence of not considering the severity of CVD during the survey. Nonetheless, the overarching trend was clear: most images, including textbook figures, became significantly more visible after enhancement, and students overwhelmingly endorsed their classroom use.

Comparison with prior enhancement studies contextualizes these results. Chen and Mo (2023) employed a Swin Transformer to achieve ΔC values near 90, enabling universal recognition of Ishihara plates, but at the cost of a complex deep network and a higher risk of color distortions. Seo’s CUD-Net produced highly ranked outputs for object distinguishability, yet required considerable computational resources (Seo et al., 2021). Pendhari et al. (2024) showed that autoencoders could recolor images to improve digit readability while supporting subsequent classification. While producing slightly lower maximum ΔC than Chen’s work (Chen and Mo 2023), the present approach retained interpretability, computational simplicity, and minimal color distortion, with ΔE values well within perceptual tolerance.

Recent comparative studies have strengthened confidence in digital and psychophysical approaches to color vision assessment. Klinke et al. (2024) demonstrated that presenting Ishihara plates on calibrated PC monitors or smartphones yielded comparable outcomes, with sensitivity of 94.4 and 96.0% and specificity of 82.4 and 94.7%, respectively, showing no significant difference between display modes. Fanlo-Zarazaga et al. (2024) validated the DIVE Color Test against both Ishihara and Farnsworth–Munsell 100-Hue, reporting perfect agreement with Ishihara in detecting CVD (Cohen’s *κ* = 1.00) and a strong correlation with Farnsworth (*ρ* = 0.80), while also quantifying severity thresholds in ΔE units (ICC = 0.83 for repeatability). Parente et al. (2025) compared the Colour Assessment and Diagnosis (CAD) test with the Cambridge Colour Test (CCT) in 66 participants and found statistically equivalent vector lengths and ellipse orientations, concluding that CAD and CCT are complementary tools for quantifying severity. Collectively, these studies show that both display-based and psychophysical measures provide robust, quantitative parameters, supporting our ΔE-based optimization (ΔE ≈ 4.9, clipping fraction <0.002) and CNN classification approach as clinically meaningful analogues for detecting and enhancing protanopia and deuteranopia.

Several limitations must be acknowledged. The Discussion text was language-edited with ChatGPT; the authors have verified all wording and take full responsibility for the content. The dataset was based on computer-generated Ishihara-style images rather than patient-derived or naturalistic images. While this ensures controlled evaluation, it does not fully capture the variability of real-world visuals. Moreover, only red-green deficiencies were addressed, leaving tritanopia unmodeled. Severity gradation was also not included, though perceptual impact often varies along a continuum. Though highly informative, the survey involved only 11–15 participants, limiting generalizability. This method does not cure the defect but simply helps the patient in recognizing the image through its processing by the intelligent algorithm. This model was tested using natural sceneries. However, it did not perform well performed in these images. Finally, occasional mixed responses suggest that the algorithm may not universally optimize all image types, and further refinement will be necessary for complex or organic figures.

Despite these constraints, this work’s clinical and educational relevance is considerable. Early identification would allow families and educators to implement accommodations before academic performance is affected. Strong survey responses validated the enhancement algorithm. Because the method is lightweight and computationally efficient, it can be integrated into mobile devices, classroom projectors, or e-learning platforms for real-time use. We have also tested this model on natural sceneries and images from MBBS books. However, this model did not well performed in these images.

Future work should extend evaluation to real-world photographs and diverse educational materials, incorporate tritanopia and severity grading, and recruit larger and more diverse cohorts for validation. Adaptive algorithms that tailor enhancement strength to specific content or individual perceptual responses may further improve outcomes. Ultimately, mobile application or embedded classroom tool deployment could combine real-time classifying with enhancement, creating inclusive educational environments for CVD learners.

## Conclusion

The outreach survey to schools revealed a critical gap in awareness of CVD among students, parents, and teachers. Teachers reported limited strategies for classroom adaptations. Career guidance was found inadequate: many respondents were unaware of job restrictions for individuals with CVD in India, and no systematic framework currently exists to help students navigate career choices. This underscores the need for early classifying and structured awareness programs. The One-vs-All (OvA) classification models demonstrated reliable performance in diagnosing CVDs, validating the potential of machine learning to support classifying and targeted interventions. By integrating OvA-based computational methods with survey insights, the study highlights the importance of advancing diagnostic technologies. The findings indicate that addressing CVD requires a dual approach: developing accurate computational tools for detection and translating these tools into educational policies and career guidance practices that empower students.

## Data Availability

Publicly available datasets were analyzed in this study. This data can be found at: https://www.kaggle.com/datasets/dupeljan/ishihara-blind-test-cards.

## Footnotes

^1^ https://www.kaggle.com/datasets/dupeljan/ishihara-blind-test-cards

## Glossary

CNN: Convolutional Neural Network
CVD: Color Vision Deficiency
De: Deuteranopia
IEC: International Electrotechnical Commission
ML: Machine Learning
MLP: Multilayer Perceptron
OvA: One-vs-All (classification strategy)
PCA: Principal Component Analysis
Pr: Protanopia
RGB: Red, Green, Blue (color space)
sRGB: Standard Red, Green, Blue (IEC 61966–2-1 standard)
SVM: Support Vector Machine
SVM-RBF: Radial Basis Function Support Vector Machine
α: Enhancement strength parameter (0 ≤ *α* ≤ 1.5)
$\alpha_{\mathrm{Pr}}^{*}$ , $\alpha_{\mathrm{De}}^{*}$: Optimal α values for protanopia and deuteranopia
$\beta_{\text{clip}}$: Penalty weight for pixel clipping
$\Delta C_{\mathrm{CVD}}$: Change in contrast under simulated CVD
$\Delta E_{\mathrm{norm}}$: Mean CIE76 color difference under normal vision
E: Error signal (difference between reference and deficient image)
$f_{\text{clip}}$: Fraction of pixels clipped at channel limits
$I_{\mathrm{def}}$: Simulated CVD (deficient) image
$I_{\mathrm{enh}}$: Enhanced image
$I_{\mathrm{lin}}$: Linear RGB image
$I_{\text{sRGB}}$: Reference sRGB image
L, M, S: Long-, Medium-, and Short-wavelength cones
L*, a*, b*: CIELAB color space channels
$L_{k}$: Individual channel component in CIELAB images
$\lambda_{\mathrm{nat}}$: Penalty weight for perceptual naturalness
$\mu_{j}$ , $\sigma_{j}$: Mean and standard deviation of feature *j*
N: Number of pixels in the image
S(α): Objective function for enhancement optimization
$X_{\text{fused}}$: Concatenated feature vector (ResNet + EfficientNet + DenseNet)
$X_{\mathrm{PCA}}$: PCA-transformed feature set
