# Supplementary material for: Enhancing Ishihara and educational images using machine learning: toward accessible learning for colorblind individuals
Source: Front Artif Intell. 2025 Oct 17;8:1676644. doi: 10.3389/frai.2025.1676644 (PMC12575240; doi:10.3389/frai.2025.1676644)
Supplement: Supplementary file 1 [file Table_1.DOCX]

**Supplementary information 1**

**Outreach screening dataset (28 Gujarat schools; n = 10,337; CVD positives = 121; Dec 2023–Jul 2025)**

| Sr.No | Date | School (Gujarat, India) | No.Of Student | Normal Student | Color blind Positive Student |
| --- | --- | --- | --- | --- | --- |
| 1 | 12/11/2023 | Rampura School No.32 | 130 | 128 | 2 |
| 2 | 12/18/2023 | Principal School No.33 | 238 | 234 | 4 |
| 3 | 12/19/2023 | Little Hearts School | 131 | 131 | 0 |
| 4 | 12/28/2023 | Khodiyar Nagar Prathmik School -Jol | 128 | 126 | 2 |
| 5 |  | Faculty Teacher's List | 60 | 60 | 0 |
| 6 | 1/3/2024 | K.M.Patel Balshala (Mogri) | 115 | 113 | 2 |
| 7 | 1/9/2024 | Nagar Prathmik Shala No.14 | 231 | 227 | 4 |
| 8 | 1/11/2024 | Nagar Prathmik Shala No.4 | 206 | 202 | 4 |
| 9 | 1/18/2024 | Nagar Prathmik Shala No.7 | 262 | 256 | 6 |
| 10 | 1/24/2024 | Anupam Mission School | 310 | 305 | 5 |
| 11 | 1/25/2024 | Anupam Mission School | 351 | 347 | 4 |
| 12 | 1/27/2024 | Anupam Mission School | 220 | 218 | 2 |
| 13 | 1/30/2024 | Anupam Mission School | 143 | 137 | 6 |
| 14 | 3/13/2024 | Mogari Kanya Shala | 244 | 244 | 0 |
| 15 | 3/15/2024 | Nagar Prathmik Shala No.10 | 48 | 48 | 0 |
| 16 | 3/15/2024 | Nagar Prathmik Shala No.16 | 175 | 175 | 0 |
| 17 | 3/15/2024 | Nagar Prathmik Shala No.13 | 173 | 170 | 3 |
| 18 | 3/16/2024 | Nagar Prathmik Shala No.20 | 190 | 190 | 0 |
| 19 | 3/16/2024 | Nagar Prathmik Shala No.19 | 142 | 135 | 7 |
| 20 | 7/8/2024 | Nagar Prathmik Shala No.27 | 163 | 163 | 0 |
| 21 | 7/8/2024 | Nagar Prathmik Shala No.28 | 237 | 237 | 0 |
| 22 | 7/9/2024 | Nagar Prathmik Shala No.29 | 107 | 104 | 3 |
| 23 | 7/9/2024 | Nagar Prathmik Shala No.31 | 211 | 210 | 1 |
| 24 | 7/10/2024 | Nagar Prathmik Shala No.3 | 146 | 143 | 3 |
| 25 | 7/10/2024 | Nagar Prathmik Shala No.6 | 242 | 239 | 3 |
| 26 | 9/26/2024 | Ambalal Balshala | 1543 | 1533 | 10 |
| 27 | 11/23/2024 | D.V.Patel High School,Ashi | 244 | 243 | 1 |
| 28 | 12/24/2024 | D.N.High School ,Anand | 1622 | 1584 | 38 |
| 29 | 2/7/2025 | Pioneer High School | 102 | 102 | 0 |
| 30 | 2/23/2025 | Shri B D Mehta Arasuri Kanya Vidyalaya Ambaji | 198 | 198 | 0 |
| 31 | 4/20/2025 | S.V.P.High School Kalsar | 19 | 19 | 0 |
| 32 | 7/23/2025 | D.N.High School ,Anand | 2006 | 1995 | 11 |
|  |  | TOTAL | 10337 | 10216 | 121 |
