# Supplementary material for: Enhancing Ishihara and educational images using machine learning: toward accessible learning for colorblind individuals
Source: Front Artif Intell. 2025 Oct 17;8:1676644. doi: 10.3389/frai.2025.1676644 (PMC12575240; doi:10.3389/frai.2025.1676644)
Supplement: Supplementary file 2 [file Table_2.DOCX]

Supplementary information 2

Extended literature review on CVD diagnosis and image enhancement (Tables S2.1–S2.3 and bibliography)

Table S2.1 Literature review on application different machine learning techniques in diagnosis color blindness

| **Domain / Application** | **Dataset / Stimulus** | **Methodology / Approach** | **Quantitative Outcomes / Key Findings** | **Reference** |
| --- | --- | --- | --- | --- |
| CVD diagnosis using Ishihara plates | Digital Ishihara images | CNN-based spatial and chromatic pattern analysis | High-accuracy classification of CVD type | ^[1]^ |
| Multiplatform CVD testing | Digital Ishihara templates | Cosine similarity between user response and correct template | Robust cross-platform performance | ^[2]^ |
| Real-time mobile CVD detection | Mobile camera input | KNN classification of captured colors | Reliable detection under variable lighting | ^[3]^ |
| Mobile colorimetric panel analysis | Mobile-acquired color panel images | Ensemble Bagged Trees, KNN | Accuracies of 96.1% and 97.6%, respectively | ^[4]^ |
| Ophthalmic CVD detection in retinopathy | Retinal fundus images | SVM classification | >85% accuracy | ^[5]^ |
| EEG-based CVD diagnosis | Brain responses to color stimuli | ML classification of EEG biomarkers | Differentiated CVD from color-normal subjects | ^[6]^ |
| Mobile real-time image enhancement | Digital images | LMS Daltonization | Validated by users and optometrists | ^[7]^ |
| Iterative Daltonization | Digital images | Successive hue correction passes | Improved perceptual separation | ^[8]^ |
| Personalized image enhancement | Psychophysical evaluation | Perceptual gamut mapping | Tailored corrections for individual CVD profiles | ^[9]^ |
| Personalized enhancement | Digital images | Type- and severity-specific adaptations | Maximized chromatic contrast & naturalness | ^[10]^ |
| Image enhancement via deep learning | Perceptual datasets | Deep Correct encoder–decoder | Enhanced chromatic contrast without oversaturation | ^[11]^ |
| GAN-based CVD simulation & enhancement | Digital images | GAN-generated corrections | Personalized correction and simulation | ^[12]^ |
| CVD enhancement | Digital images | Fuzzy C-means clustering + ΔE optimization | Higher subjective preference over traditional methods | ^[13]^ |
| Optimization-based enhancement | Digital images | Dual-parameter optimization (contrast & naturalness) | Balanced ΔE improvements and perceptual quality | ^[14]^ |
| Image preprocessing for enhancement | Digital images | Bicubic downsampling | Retained 78.9% color histogram similarity | ^[15]^ |
| Feature extraction | Various | LAB color space | Effective for pigment localization & perceptual difference modeling | ^[16]^ |
| Feature extraction | Various | LAB color space | Improved perceptual feature modeling | ^[17]^ |
| Segmentation & classification | Ophthalmic/dermatology images | YCbCr + hybrid histograms + SVM/LogReg | Significant accuracy improvement | ^[18]^ |
| Segmentation | Medical images | YCbCr color space | Accurate chromatic segmentation | ^[19]^ |
| Multisensory assistive tech | VR color–audio mapping | VR platform with auditory substitution | Enabled chromatic distinction via sound | ^[20]^ |
| AR assistive application | Real-world objects | AR + text-to-speech | 85% real-time color identification accuracy | ^[21]^ |
| Wearable assistive tech | Google Glass | Hands-free color recognition | Dynamic environment compatibility | ^[22]^ |
| Ophthalmic CVD detection | Retinal images | SVM, XGBoost | 92% accuracy | ^[23]^ |
| Perceptual quality evaluation | Digital images | Dual-parameter evaluation (ΔE + naturalness) | Maintained visibility for CVD & naturalness for normals | ^[24]^ |

Table S2.2 Different techniques and achieved accuracies in color blind studies

|  | Context | Technique / Model | Quantitative results (as reported) | Notes relevant to Ishihara perception/enhancement | Reference |
| --- | --- | --- | --- | --- | --- |
| 1 | Image enhancement (general) | ESRGAN paired with CNN backbones (MobileNetV2, VGG19, ResNet152V2) | 91% (ESRGAN+MobileNetV2); 86% (ESRGAN+VGG19); 96% (ESRGAN+ResNet152V2) | Strongest accuracy with ResNet152V2 suggests deeper backbones can yield better perceptual improvement pipelines for Ishihara-type images. | ^[25]^ |
| 2 | Color-vision support | Iterative daltonization | Outperforms non-iterative methods (no specific % reported) | Iterative scheme avoids mapping imperceptible colors into already similar hues, improving color separability for CVD users viewing Ishihara images. | ^[8]^ |
| 3 | CVD vs. normal vision during Ishihara test (EEG) | KNN classifier | KNN: Accuracy 85.2% | Closest to the target task; no Random Forest (RF) metric reported. | ^[26]^ |
| 4 | CVD diagnosis (EEG-SSVEP with Ishihara context) | KNN among classifiers | KNN used; no RF metric reported | CVD-focused pipeline; lacks RF numbers for comparison. | ^[27]^ |

Table S2.3 Role of artificial intelligence in improving diagnostic accuracy and consistency in diagnosing color vision defects is influenced by ambient lighting conditions, the type of test administered, the angle at which the test is presented, correction of refractive errors, and pathological issues affecting the eye’s structure.

| **Factor** | **Challenge** | **AI Solution** | **Reference** |
| --- | --- | --- | --- |
| Ambient Lighting Conditions | Color discrepancies due to lighting variability | Color calibration using AI models  *AI can normalize images across different lighting environments to maintain consistency.* | ^[28]^ |
| Type of Test Administered | Variability in test results across methods | Standardization and optimization of test protocols  *Machine learning can harmonize outputs from diverse test types to reduce variability.* | ^[29]^ |
| Angle of Test Presentation | Skewed or inconsistent results due to test angle | Geometric correction through CNN-based models  *CNNs can detect and correct skewed test presentations to ensure accurate interpretation.* | ^[30]^ |
| Correction of Refractive Errors | Reduced accuracy from uncorrected refractive errors | Automated refractive error correction via AI analysis  *Deep learning can estimate and correct refractive errors before test analysis.* | ^[31]^ |
| Pathological Issues | Eye diseases influencing color vision outcomes | AI-assisted detection and differentiation of ocular pathologies  *AI imaging helps distinguish between true color vision deficiency and disease-related vision loss.* | ^[32]^ |

Bibliography

1. Dhawale K, Vohra A, Jain P, Kumar T. A Framework to Identify Color Blindness Charts Using Image Processing and CNN [Internet]. In: Communications in Computer and Information Science. 2021. page 447–56.Available from: https://www-scopus-com.elibrary.nirmauni.ac.in/record/display.uri?eid=2-s2.0-85122538583

2. Anggraini N, Ramadhani W, Afrizal S, Saepudin D. Integration of Ishihara and Cambridge Methods for Multiplatform Color-Blindness Detection Using Cosine Similarity [Internet]. In: 2024 12th International Conference on Cyber and IT Service Management (CITSM 2024). 2024. page 1–6.Available from: https://www-scopus-com.elibrary.nirmauni.ac.in/pages/publications/85214912887

3. Widianto E, Lutfian K, Safitri A, Nugroho A. A Mobile Application CODEC (Color Detection) for Color-blind People using KNN [Internet]. In: Proceedings of the 2023 8th International Conference on Informatics and Computing (ICIC 2023). 2023. page 155–60.Available from: https://www-scopus-com.elibrary.nirmauni.ac.in/record/display.uri?eid=2-s2.0-85183461106

4. Tania M, Lwin K, Shabut A, Hossain M. Clustering and Classification of a Qualitative Colorimetric Test [Internet]. In: Proceedings of the 2018 International Conference on Computing, Electronics and Communications Engineering (iCCECE 2018). 2018. page 83–8.Available from: https://www-scopus-com.elibrary.nirmauni.ac.in/record/display.uri?eid=2-s2.0-85063424299

5. Chanda P, Sarkar S. Medical Image Based Approach For Classification Of Several Stages For Retinopathy Disease Using Machine Learning Methodology [Internet]. In: IET Conference Proceedings. 2020. page 404–8.Available from: https://www-scopus-com.elibrary.nirmauni.ac.in/record/display.uri?eid=2-s2.0-85174653446

6. Hamid MA, Singh J. EEG Signal Processing for Detection of Colour Vision Deficiencies. ECS Trans 2022;107(1):10053.

7. Tecson G, Cayabyab G, Calanda F, Reyes F. Covisance: A real time mobile recolorization tool for aiding color vision deficient users utilizing D-15 color arrangement test [Internet]. In: ACM International Conference Proceeding Series. 2017. Available from: https://www-scopus-com.elibrary.nirmauni.ac.in/record/display.uri?eid=2-s2.0-85042067197

8. Tasnim A, Hasan M. An improved dynamic daltonization for color-blinds [Internet]. In: 5th IEEE Region 10 Humanitarian Technology Conference 2017, R10-HTC 2017. 2018. page 801–6.Available from: https://www-scopus-com.elibrary.nirmauni.ac.in/record/display.uri?eid=2-s2.0-85047407881

9. Bento-Torres NVO, Rodrigues AR, Côrtes MIT, Bonci DM de O, Ventura DF, Silveira LC de L. Psychophysical Evaluation of Congenital Colour Vision Deficiency: Discrimination between Protans and Deutans Using Mollon-Reffin’s Ellipses and the Farnsworth-Munsell 100-Hue Test. PLoS One 2016;11(4):e0152214.

10. Kereste s N, \DJur\djevi’c S, Novakovi’c D, Vladi’c G. Customized daltonization: Adaptation of different image types for observers with different severities of color vision deficiencies. Universal Access in the Information Society 2023;23(1):43–58.

11. Petrovi’c G, Fujita H. Deep Correct: Deep learning color correction for color blindness [Internet]. In: Frontiers in Artificial Intelligence and Applications. 2017. page 379–86.Available from: https://www-scopus-com.elibrary.nirmauni.ac.in/record/display.uri?eid=2-s2.0-85101296938

12. Gangwani G, Ragupathy A, Anand N, Srinivas K. Examining Strategies for Correcting Color Vision Deficiency: A Survey [Internet]. In: 2024 4th IEEE International Conference on Software Engineering and Artificial Intelligence (SEAI 2024). 2024. page 86–91.Available from: https://www-scopus-com.elibrary.nirmauni.ac.in/pages/publications/85205971924

13. Singla A, Meenakshi M. A Fuzzy based naturalness preserving image color compensation method for colorblindness [Internet]. In: Proceedings of the IEEE International Conference on Image Information Processing. 2021. page 330–5.Available from: https://www-scopus-com.elibrary.nirmauni.ac.in/record/display.uri?eid=2-s2.0-85125868935

14. Zhu Z, Toyoura M, Go K, Mao X. Naturalness- and information-preserving image recoloring for red–green dichromats. Signal Processing: Image Communication 2019;77:162–71.

15. Setiawan A A, Widodo, AM, Firmansyah, G, Wisnujati. Network Intrusion Detection Using 1D Convolutional Neural Networks. Proceedings - ICE3IS 2024 [Internet] 2024;Available from: https://www-scopus-com.elibrary.nirmauni.ac.in/record/display.uri?eid=2-s2.0-85215128377&origin=scopusAI

16. Uçar A. Color face recognition based on curvelet transform [Internet]. In: Proceedings of the 2012 International Conference on Image Processing, Computer Vision, and Pattern Recognition (IPCV 2012). 2012. page 1–5.Available from: https://www-scopus-com.elibrary.nirmauni.ac.in/record/display.uri?eid=2-s2.0-84873307442

17. Jabal M, Abdullah A, Najjar F, Manan W. A Novel Color Feature for the Improvement of Pigment Spot Extraction in Iris Images. Journal of Image and Graphics (United Kingdom) 2024;12(2):47–53.

18. Agrawal S, Verma N, Tamrakar P, Sircar P. Content based color image classification using SVM [Internet]. In: Proceedings of the 2011 8th International Conference on Information Technology: New Generations (ITNG 2011). 2011. page 902–7.Available from: https://www-scopus-com.elibrary.nirmauni.ac.in/record/display.uri?eid=2-s2.0-80051506482

19. Das A, Ghoshal D. Human Skin Region Segmentation Based on Chrominance Component Using Modified Watershed Algorithm. Procedia Computer Science 2016;85:354–62.

20. Seefried E, Bahny J, Jung C, Arefin M. Perceiving and Learning Color as Sound in Virtual Reality [Internet]. In: Proceedings of the 2024 IEEE International Symposium on Mixed and Augmented Reality Adjunct (ISMAR-Adjunct 2024). 2024. page 250–5.Available from: https://www-scopus-com.elibrary.nirmauni.ac.in/record/display.uri?eid=2-s2.0-85214357332

21. Irawati I, Muawwal A, Darwis H, Hayati L. Implementation of Augmented Reality, TTS, and Midpoint Algorithm in Supporting People with Color Vision Deficiency [Internet]. In: Proceedings of the 2nd East Indonesia Conference on Computer and Information Technology (EIConCIT 2018). 2018. page 323–7.Available from: https://www-scopus-com.elibrary.nirmauni.ac.in/record/display.uri?eid=2-s2.0-85074881165

22. Fuller T, Sadovnik A. Image level color classification for colorblind assistance [Internet]. In: Proceedings of the International Conference on Image Processing (ICIP). 2017. page 2831–5.Available from: https://www-scopus-com.elibrary.nirmauni.ac.in/record/display.uri?eid=2-s2.0-85045297517

23. Tuncer H, Altinel D. Classification of Eye Diseases using Machine Learning with Preprocessing [Internet]. In: 8th International Artificial Intelligence and Data Processing Symposium (IDAP 2024). 2024. page 312–7.Available from: https://www-scopus-com.elibrary.nirmauni.ac.in/record/display.uri?eid=2-s2.0-85207935297

24. Zhao S, Zhao X, Dai Q. A dual-parameter evaluation method for color-enhancement technologies and its application to lighting-spectrum optimization for color-deficient observers. Optics Communications 2024;535:129275.

25. Tussupov J, Kozhabai K, Bayegizova A, Kassenova L, Manbetova Z, Glazyrina N, et al. Applying machine learning to improve a texture type image. Eastern-European Journal of Enterprise Technologies 2023;2(2(122)):13–8.

26. Ekhlasi A, Ahmadi H, Molavi A, Saadat Nia M, Motie Nasrabadi A. EEG signal analysis during Ishihara’s test in subjects with normal vision and color vision deficiency. Biomedical Physics & Engineering Express 2021;7(2):025008.

27. AlEssa GN, Alzahrani SI. EEG-Based Methods for Diagnosing Color Vision Deficiency: A Comprehensive Review. Applied Sciences 2024;14(17):7579.

28. Tiwari B, Mittal A, Kaur N. Calibration card based technique for accurate color representation in clinical settings. Measurement 2025;249:116816.

29. Martucci A, Gallo Afflitto G, Pocobelli G, Aiello F, Mancino R, Nucci C. Lights and Shadows on Artificial Intelligence in Glaucoma: Transforming Screening, Monitoring, and Prognosis. Journal of Clinical Medicine 2025;14(7):2139.

30. Jamali Dogahe S, Garmany A, Sadegh Mousavi S, Khanna CL. Predicting 60-4 visual field tests using 3D facial reconstruction. Br J Ophthalmol 2023;108(1):112–6.

31. Nguyen T, Ong J, Jonnakuti V, Masalkhi M, Waisberg E, Aman S, et al. Artificial intelligence in the diagnosis and management of refractive errors. European Journal of Ophthalmology 2025;35(4):1456–80.

32. Rampat R, Deshmukh R, Chen X, Ting DSW, Said DG, Dua HS, et al. Artificial Intelligence in Cornea, Refractive Surgery, and Cataract: Basic Principles, Clinical Applications, and Future Directions. Asia Pac J Ophthalmol (Phila) 2021;10(3):268–81.
