# Supplementary material for: Enhancing Ishihara and educational images using machine learning: toward accessible learning for colorblind individuals
Source: Front Artif Intell. 2025 Oct 17;8:1676644. doi: 10.3389/frai.2025.1676644 (PMC12575240; doi:10.3389/frai.2025.1676644)
Supplement: Supplementary file 3 [file Table_3.DOCX]

# Supplementary information 3

**Mathematical derivations for the simulation and enhancement pipeline (Eqs. 1–11: sRGB↔LMS, CVD simulation, daltonization objective)**

# Mathematical Derivation Eqs. (1) to (11)

*Eq. (1) sRGB to linear RGB (inverse transfer)*

Goal is to map display encoded sRGB $s\in[0,1]$ to linear light $l\in[0,1]$.

- Assume a linear toe with slope $A$ and displaced power law with exponent $\gamma$.
  - Low branch: $l=As$
  - High branch: $l=\left( s+\frac{C}{B} \right)^{\gamma}$
- Constants defined by sRGB standard (IEC 61966-2-1, 1999): $\gamma=2.4$, $A=$1/12.92, $B=$1.055, $C=$-0.055.
- To find breakpoint from value continuity: At the join $s_{b}$,
  - $As_{b}=\left( s_{b}+\frac{C}{B} \right)^{\gamma}$
  - Solve with given constants, resulting $s_{b}=$0.04045
  - Corresponding linear breakpoint $l_{b}=As_{b}=$0.0031308.

Therefore,

| $I_{lin}\left( s \right)=\left\{ \begin{aligned} \frac{s}{12.92} s\leq0.04045 \\ \left( \frac{s+0.055}{1.055} \right)^{2.4}, s>0.0405 \end{aligned} \right.$ | (1) |
| --- | --- |

**Eqs. (2) and (3): linear RGB to LMS**

Goal is to map linear RGB to cone responses LMS

Step 1: RGB to XYZ (from primaries) (IEC 61966-2-1, 1999)

Use sRGB primaries ($x_{r}$,$y_{r}$), ($x_{g}$,$y_{g}$), ($x_{b}$,$y_{b}$) and white D65 ($x_{w}$,$y_{w}$). For each primary, the XYZ direction is $\left[ \frac{x}{y},1,\frac{1-x-y}{y} \right]^{T}$; solve gains ($k_{r}$,$k_{g}$, $k_{b}$) so $\left[ \begin{matrix} 1 & 1 & 1 \end{matrix} \right]^{T}$ maps to the D65 white with $Y=$1. This gives the standard $M_{RGB\to XYZ}$

Step 2: XYZ to LMS (cone fundamentals)

D65-adapted cone transfer $M_{XYZ\to LMS}$ was adopted from (Machado, Oliveira & Fernandes, 2009).

$LMS=M_{RGB\to LMS}{RGB}_{lin}$

With $M_{RGB\to LMS}=M_{XYZ\to LMS}M_{RGB\to XYZ}$

Using Machado’s cone set with sRGB: $M_{RGB\to LMS}=\left[ \begin{matrix} 0.314 & 0.639 & 0.046 \\ 0.155 & 0.757 & 0.087 \\ 0.018 & 0.109 & 0.872 \end{matrix} \right]$

Eqs. (4) and (5): LMS to simulated CVD LMS (Machado, Oliveira & Fernandes, 2009)

**Goal is to li**nearly emulate dichromacy in LMS.

- Neutrals stay neutral: each row sums to 1 (white preserved, no tint in greys).
- “Missing” cone behavior: for protanopia (L-absent), the effective L depends only on M,S (zero in L column in row 1); for deuteranopia (M-absent), the effective M depends only on L,S (zero in M column in row 2).
- Fit the remaining coefficients so confusion lines and white are respected (Machado et al., constrained least-squares against a physiology-based model).

| $M_{De}=\left[ \begin{matrix} 0.625 & 0.375 & 0.000 \\ 0.700 & 0.300 & 0.000 \\ 0.000 & 0.300 & 0.700 \end{matrix} \right]$ | (4) |
| --- | --- |
|  |  |
| $M_{Pr}=\left[ \begin{matrix} 0.567 & 0.433 & 0.000 \\ 0.558 & 0.442 & 0.000 \\ 0.000 & 0.242 & 0.758 \end{matrix} \right]$ | (5) |
|  |  |

**Eq. (6) linear RGB to sRGB (forward transfer)** (IEC 61966-2-1, 1999)

Goal is to map linear light $l$ back to sRGB $s$.

Same constants as Eq. (1): $\gamma=2.4$, $A=$1/12.92, $B=$1.055, $C=$-0.055

| $sRGB\left( l \right)=\left\{ \begin{aligned} 12.92 l l\leq0.0031308 \\ 1.055l^{\frac{1}{2.4}}-0.055, l>0.0031308 \end{aligned} \right.$ | (6) |
| --- | --- |

The function is value continuous at $l_{b}$; the tiny slope mismatch is as in the IEC specification.

**Eqs. (7) and (8) Error feedback daltonization** (Simon-Liedtke et al., 2017; Farup, 2020)

Let $D(.)$ be the full CVD simulation pipeline (sRGB to linear to LMS to Mdef to linear to sRGB)

Error signal (Eq. 7)

$E=I_{sRGB}-I_{def}$, where $I_{def}=D(I_{sRGB})$

Idea: linearize $D\left( I+\Delta\right)\approx D\left( I \right)+J(\Delta)$. A first order step that pulls $D\left( I+\Delta\right)$ toward $I$ minimizes $\left\| D\left( I+\Delta\right)-I \right\|^{2}$, whose gradient at $\Delta=0$ is proportional to $I-D\left( I \right)$.

| $I_{enh}=clip(I_{sRGB}+\alpha.E,0,1)$ | (8) |
| --- | --- |

Equations (9)–(11) (Simon-Liedtke et al., 2017; Farup, 2020; CIE 1976)define the objective function for optimizing the enhancement parameter (α) in image daltonization for color vision deficiency (CVD). The derivation is from first principles, explaining each term and linking them to standards such as IEC sRGB and CIE L*a*b* (CIELAB).

## Equation (9): Objective Function

We aim to maximize perceptual contrast for simulated CVD observers while penalizing unnatural deviations under normal vision and avoiding channel clipping. The constrained optimization is expressed as:

maximize $C_{CVD}(I_{enh})$

subject to $D_{norm}$ ≤$\tau_{nat}$ , $f_{clip}=$ 0

Rewriting with penalty terms yields:

| $S\left( \alpha\right)=\Delta C_{CVD}-\lambda_{nat}\Delta E_{norm}-\beta_{clip}f_{clip}$ | (9) |
| --- | --- |

Here:
- $C_{CVD}$: change in CVD contrast.
- $\Delta E_{norm}$: mean perceptual color difference (naturalness penalty).
- $f_{clip}$: fraction of saturated pixels.
- $\lambda_{nat}$ and $\beta_{clip}$: weights controlling penalties (e.g., $\lambda_{nat}$ = 0.15, $\beta_{clip}$ = 0.05).

## Equation (10): Change in CVD Contrast

Let the CVD-simulated image be represented in CIELAB with pixel vectors $x_{p}=\left[ \begin{matrix} L_{p}^{*} & a_{p}^{*} & b_{p}^{*} \end{matrix} \right]$. Define the sample mean $\mu$ and covariance $\Sigma$ as:

$\mu=\frac{\sum x_{p}}{N}$, $\Sigma=\frac{\sum_{p}^{N} \left( x_{p}-\bar{x} \right)\left( x_{p}-\bar{x} \right)^{T}}{N}$

The total spread is given by the trace of $\Sigma$, which equals the sum of variances across channels:

$tr(\Sigma) = Var(L*) + Var(a*) + Var(b*)$

Thus, the gain in contrast from enhancement is:

| $\Delta C_{CVD}=\left[ \sum_{k=1}^{3} Var\left( L_{k}^{corr} \right) \right]-\left[ \sum_{k=1}^{3} Var\left( L_{k}^{orig} \right) \right]$ | (10) |
| --- | --- |

## Equation (11): Naturalness Penalty

Perceptual deviation under normal vision is measured using the CIE76 color difference (${\Delta E}_{76}$) between original and enhanced images:

${\Delta E}_{76}\left( p \right)=\sqrt{{({\Delta L}_{p}^{*})}^{2}+{({\Delta a}_{p}^{*})}^{2}+{({\Delta b}_{p}^{*})}^{2}}$

Averaging across all N pixels gives Eq. (11):

## References

CIE 1976.CIE 1976 UCS Diagram /∆E*94 Color difference formula (CIE 1994) - Part IV - Precise Color Communication | KONICA MINOLTA. *Available at* *https://www.konicaminolta.com/instruments/knowledge/color/part4/08.html* (accessed September 8, 2025).

Farup I. 2020. Individualised Halo-Free Gradient-Domain Colour Image Daltonisation. *Journal of Imaging* 6:116. DOI: 10.3390/jimaging6110116.

IEC 61966-2-1. 1999. Multimedia systems and equipment – Colour measurement and management – Part 2-1: Colour management – Default RGB colour space – sRGB: Amendment 1.

Machado GM, Oliveira MM, Fernandes LAF. 2009. A Physiologically-based Model for Simulation of Color Vision Deficiency. *IEEE Transactions on Visualization and Computer Graphics* 15:1291–1298. DOI: 10.1109/TVCG.2009.113.

Simon-Liedtke J, Flatla DR, Bakken EN, Flatla DR, Bakken EN. 2017. Checklist for Daltonization methods: Requirements and characteristics of a good recolouring method. *Electronic Imaging* 29:21–27. DOI: 10.2352/ISSN.2470-1173.2017.18.COLOR-029.
